# In vitro effect of *Mikania cordata* leaf extracts on wound healing

**DOI:** 10.1186/s12906-025-05110-7

**Published:** 2025-10-09

**Authors:** Naomi Navaneeta Vijithsingh, Kaushalya Anuradha, Shiroma Mangalika Handunnetti, Narmada Fernando, Kanishka Senarath

**Affiliations:** 1 Institute of Biochemistry, Molecular Biology and Biotechnology, University of Colombo, No. 90, Cumarathunga Munidasa Mawatha, Colombo 03, Colombo, Sri Lanka; 2 Department of Pharmacology and Toxicology, Medical College of Georgia, Augusta University, Augusta, GA USA

**Keywords:** *Mikania cordata*, * In vitro* scratch assay, Wound healing, Endothelial cells, Vascular endothelial growth factor, Nitric oxide

## Abstract

**Backgroud:**

*Mikania cordata* is a resource-rich plant which is used in traditional medicinal practices to treat eye sores, snake and scorpion bites, cough, a range of gastrointestinal disorders and topical applications for cuts, wounds and to stop bleeding. This study examined the in vitro wound healing activity of aqueous, acetone and methanolic extracts of *M. cordata* leaves.

**Methods:**

Human macro-vascular endothelial cells (EC) were exposed to the three extracts at 3.9–500 µg/mL to determine their non-toxic concentrations and in vitro wound healing ability was assessed using scratch assay. The nitrite levels produced by scratched ECs were assessed by Griess assay and the gene expression of vascular endothelial growth factor (VEGF) at 48 h was assessed using the conventional PCR by quantifying the intensity of gel band.

**Results:**

All concentrations of the three extracts of *M. cordata* tested were non-toxic to EC. Methanolic extract and positive control Allantoin showed the highest in vitro wound healing activity of 75% and 82% compared to that by aqueous (66%) and acetone (56%) extracts (*p* < 0.001). EC treated with 125 µg/ml of methanolic extract showed a significant increase in nitrite level when compared to that of Allantoin ( *p* < 0.001). A similar expression of VEGF (0.44) was observed in EC treated with methanolic extract of *M. cordata* when compared to that of the EC treated with Allantoin (0.42).

**Conclusion:**

*M. cordata* leaves are potent in wound healing, with methanolic extract showing the highest activity. These findings demand further studies to investigate the active compounds present in *M. cordata* which specifically contribute to wound healing and the mechanisms induced to promote wound healing.

**Supplementary Information:**

The online version contains supplementary material available at 10.1186/s12906-025-05110-7.

## Introduction

Traditional medicine referred to as the practices, skills, and knowledge that depends on the indigenous experiences, theories, and beliefs can be used effectively for the maintenance of health, diagnosis, prevention, and in treating illnesses [1]. Although the development and the production of chemically synthesized drugs in healthcare have been revolutionized in most parts of the world, a large population in developing countries still rely on herbal medicines and traditional practitioners for primary healthcare [2]. A very high proportion of the population in Africa (90%) and India (70%) rely on the use of herbal medicine for their needs in health care [2]. Around 80% of the world’s population is estimated to use traditional medicine [3].

In Sri Lanka, a large proportion of the population has been practicing traditional medicine over the past 3000 years and this traditional treatment can be categorized into four medical systems such as Ayurveda, Siddha, Unani, and Deshiya Chikitsa [4]. Around 60–70% of the rural population in Sri Lanka depend on natural and traditional medicine for primary healthcare [5]. Medicinal plants play a vital role in the prevention of diseases in traditional medicine. However, much effort is needed for the proper identification and recognition of these medicinal plants for designing and implementing such dignostic or therapeutic strategies. Also, utilizing medicinal plants in the traditional medicine to treat several incurable diseases while conserving and efficiently using the medicinal plants with proper maintenance of biodiversity is essential [5].

Wound healing is considered as a series of interconnected biochemical and cellular changes with multiple stages. In adult humans, optimal wound healing involves the following events: (1) rapid hemostasis; (2) appropriate inflammation; (3) mesenchymal cell differentiation, proliferation, and migration to the wound site; (4) suitable angiogenesis; (5) prompt re-epithelialization over the wound [6]. Wound healing is a complex process that is heavily influenced by the nature and degree of the injury. Angiogenesis, the process of creating new blood vessels, is a crucial event during the proliferation stage [7]. An angiogenic growth factor could facilitate the healing of long-term wounds with reduced vascularity and hypoxia. Vascular endothelial growth factor (VEGF) is one such potential. It can promote vascular permeability, function as a chemotactic agent, and endothelial cell mitogen [8]. There have been descriptions of other angiogenic growth factors, such as transforming growth factor β (TGF-β) and basic fibroblast growth factor (bFGF), but VEGF is distinct as it affects several elements of the wound healing cascade, such as angiogenesis and more recently epithelization and collagen deposition [8, 9].

Antibiotics, antiseptics, and desloughing agents help wound healing, albeit most of these synthetic treatments have limitations due to side effects [10]. Therefore, medicinal plants with wound healing aspects and with lesser side effects would be important in view of these limitations. Hence, investigations on medicinal plants, combinations of plants or extracts are needed to be scientifically validated to develop new or improved wound healing remedies.

Medicinal plants have been shown in several studies to speed up the recovery time of wound healing by reducing bacterial count, improving of collagen deposition and by elevating the levels of fibroblasts and fibrocytes [11] and to promote wound healing in diabetic, infected, and open wounds [10]. *Mikania cordata* is a medicinal plant that has various uses in treatment for coughs, swellings, wounds, dysentery, itching [12, 13].

*M. cordata* is a perennial vine that grows quickly on the soil surface or on a tree and is a member of the family *Asteraceae.* It is commonly known as Climbing Hempweed or ‘Wathu paalu’ or ‘Gam paalu’ in Sinhala and is considered a weed in rubber plantations. It has long-petioled, deltoid-ovoid or heart-shaped leaves that are 4 to 10 cm long and have a pointy tip, rounded, heart-shaped, and toothed margins. There are four-flowered heads that are cylindrical and 6 to 9 mm long. This is spread via wind dispersal of seed and roots at nodes that are in contact with the soil [12, 14].

*M. cordata* is used to treat wounds and cuts by the Garo tribal people in Netrokana district in Bangladesh where the entire plant is ground into a paste and applied to wounds to stop the bleeding [15]. Further, the Santals tribals in the Thakurgaon district use the leaves of *M. cordata* for the treatment of cuts and wounds and also as a remedy to treat dengue fever [15]. Moreover in Jamalpur district, Bangladesh, *M. cordata* is used as poultice for treatment of swelling, itches and wounds [15]. In addition, the roots and leaves of *M. cordata* have also been reported for being used for treatment of various other illnesses and disease conditions. The juice of *M. cordata* leaves is reportedly used by native herbal practitioners in India to soothe insect and scorpion bites [13]. A decotion of *M. cordata* leaves is commonly used as a treatment of gastric ulcer in the Rajbari district of Bangladesh [16]. This study also reports on the bioactive component of *M. cordata* having anti-ulcerogenic effect [16]. The aqueous and ethanolic extracts of leaves have been shown have anti-ulcer activity, anti-diarrhoea effect and analgesic properties [17]. Aqueous and organic solvent extracts of *M. cordata* leaves have shown potent antimicrobial and hypolglycemic activities [18]. Methanolic extract of *M. cordata* leaves have shown regulation of anti-inflammatory and antioxidant responses by via the inactivation of NF-κB and MAPK signaling pathways and activation of Nrf2 in LPS-induced RAW 264.7 macrophages [19]. It is reported to have psycho-pharmacological, neuro-pharmacological, antibacterial, antifungal activities and therapeutic properties against pain, inflammation, hyperthermia, ulcer and carcinogenesis [16]. However, to date the wound healing bioactivity of *M. cordata* has not been reported or scientifically validated.

The present study was focused on investigating in vitro wound healing activity of leaves of *M. cordata* using the human endothelial cell (EC) line; EA. hy926 as an in vitro model to assess the wound healing activity of three different extracts, i.e. aqueous, acetone, and methanolic extracts. Characteristic functionality feature of the EA.hy926 includes inflammation, angiogenesis, and homeostasis/thrombosis. Hence, this particular cell line, EA. hy926 was chosen since angiogenesis which is an essential function that requires for a wound to heal is displayed by this model cell line. The scratch assay using EC was performed with the non-toxic concentrations of the three leaf extracts to determine their in vitro wound healing activity in comparison to allantoin, the positive control. The effect of *M. cordata* leaf extracts was also tested to determine the expression of VEGF, which is an important growth factor during the process of wound healing using conventional PCR.

## Materials and methods

### Chemicals and reagents

Dulbeccos Modified Eagle Medium (DMEM) (Sigma Aldrich, USA), Fetal bovine serum (FBS) (ATCC, USA), Pencillin Streptomycin (Sigma Aldrich, USA), Trypsin EDTA (Sigma Aldrich, USA), NaCl (Sigma Aldrich, USA), Na_2_HPO_4_ (Sigma Aldrich, USA), KH_2_PO_4_ (Sigma Aldrich, USA), 3-[4,5-dimethylthiazole-2-yl]−2,5-diphenyltetrazolium bromide (MTT) (Sigma Aldrich, USA), Sulforhodamine B (Sigma Aldrich, USA), Trypan Blue (Sigma Aldrich, USA), Tris Base (Sigma Aldrich, USA), Trichloroacetic acid (Sigma Aldrich, USA), Methanol (Sigma Aldrich, USA), Acetone HPLC grade (Research labs, India), Allantoin (Sigma Aldrich, USA), Agarose powder (Sigma Aldrich, USA), RNEasy mini kit Total RNA extraction kit (Qiagen, USA), Go script reverse transcriptase (Promega, USA).

### Authentication and collection of the plant

Leaves of *Mikania cordata* (Burm.f.) B.L.Rob. were collected during January 2022 from a private land located in the village called Warana (07°06′15.3″N 80°04′28.9″E) in the Gampaha district of Sri Lanka. Identification and authentication of the plant was certified by Ms. Subhanie Ranasinghe, Deputy Director of the National Herbarium, Royal Botanical Gardens, Peradeniya, Sri Lanka. The voucher specimen of *M. cordata* was deposited (NN/mc/001) at the Institute of Biochemistry, Molecular Biology and Biotechnology, University of Colombo, Sri Lanka.

### Ethical clearance

Ethics excemption to carry out the study was obtained from Medical Research Institute, Colombo 08, Sri Lanka (ERC reference Number: 27/2021).

### Cuture and maintenance of EA.hy926 cells

EA.hy926 (ATCC CRL^®^−2922 ™) human macrovascular endothelial cells were cultured in DMEM supplemented with 10% FBS and 50 U/ml Penicillin-Streptomycin solution and maintained under conditions of 5% of CO_2_ in 37 °C (CO_2_ incubator, Thermo Scientific, USA) according to the ATCC guidelines.

### Preparation of plant extracts

Aqueous extract of *M. cordata* was prepared by grinding (using a mechanical grinder) 700 g of fresh leaves with water (w/v 50%) and double filtering using a muslin cloth. A yield of 1.31% of filtrate was collected after it was freeze dried and this was stored at −20 °C until use.

The air-dried ground plant leaves (10 g) were sequentially extracted with the solvents acetone and methanol (w/v 1:10). The two extracts were filtered and concentrated using a rotary vaccum evaporator. The percentage yield of the resulting acetone and methanolic extracts was 3.12% and 5%, respectively, and was stored at −20 °C until use.

### Determination of non-toxic concentrations of M. cordata leaf extracts on ECs

Both SRB and MTT assays were performed to determine the viability and overall cell function to investigate the non-toxic concentrations of the three extracts prepared. 

SRB assay was conducted as described previously [20]. The EA.hy926 cells were seeded at a density of 5 × 10^4^ cells/well and were cultured for 48 h. EC were treated with two-fold dilutions of the three extracts of *M. cordata* ranging from 3.9 to 500 µg/ml for either 24–48 h at 37 °C in 5% CO_2_. The cells were washed with pre-warmed PBS and was fixed with Tricholoroacetic acid solution. The plates were then incubated for 1 h at 4°C. Monolayers of the cells were washed with tap water and the cells were stained with 0.2 mg/ml of SRB for 30 min at room temperature. The excess stain was removed by washing the cells with 1% glacial acetic acid. Tris-base was added and the plates were shaken for 1 h at room temperature. The absorbance was read at OD 540 nm using a microplate reader (Synergy HTX, multi-mode reader).

MTT assay was performed as described previously [21] with some modifications to determine the functionality of EC following in vitro treatment with *M. cordata* extracts. EA. hy926 cells were seeded at a density of 5 × 10^4^ cells and cultured for 48 h. EC were treated with two-fold dilutions of the three extracts of *M. cordata* ranging from 3.9 to 500 µg/ml for either 24–48 h at 37 °C in 5% CO_2_. The cells were washed once with pre-warmed PBS. MTT working solution (200 µl) was added and incubated at 37 °C in 5% CO_2_ for 4 h. The formazan crystals were dissolved in 100 µl of isopropanol containing 0.4% HCl and the absorbance of the plate was read at OD 570 nm using a microplate reader (Synergy HTX, multi-mode reader).

### In vitro assay for wound healing/scratch assay

EC were seeded at 0.2 × 10^6^ cells/ml concentration in a 24 well plate and incubated for 24 h in a 5% CO_2_ incubator at 37 °C. The ‘scratch’ was created by drawing a vertical line holding the 200 µl pipette tip at an angle of 45°. The media in the wells were aspirated and the cells were washed with 1X PBS [22]. The cells were treated with 200 µl of aqueous, methanolic and acetone extracts (each at 125, 250 and 500 µg/ml concentrations) of *M. cordata* separately and were incubated for 12, 24 and 48 h.

The morphology of cells and the closure of the ‘scratch’ was imaged at 0, 12, 24 and 48 h using a phase contrast microscope (Olympus CK40) at a magnification of 200 X. The percentage of ‘wound’/gap closure was analysed using ‘ImageJ’ by obtaining the area under the curve [22].

$$\%\ \text{ of wound closure} \;=\; \frac{\text{Initial distance of the gap} - \text{Final distance of the gap}}{\text{Initial distance of the gap}} \times 100\%$$

### Assessment of the effect of M. cordata leaf extracts on nitric oxide produced by ECs in the scratch assay

In a 24 well plate, EA.hy 926 endothelial cell were seeded at a cell number of 0.2 × 10^6^ cells/ml and was incubated for 24 h at 5% CO_2_ incubator at 37 °C. The scratch was created and the culture supernatants were aspirated after 12, 24 and 48 h and centrifuged at 10,000 *g* for 10 min. The levels of nitric oxide (NO) produced was measured by assessing the nitrite levels in culture supernatants using Griess assay [23]. A volume of 100 µl of each culture supernatant was added to an microtiter plate in triplicates. NaNO_2_ standards in the concentration of 0.195, 0.391, 0.781, 1.56, 3.125, 6.25, 12.5, 25, 50 and 100 µM were aslo added to the microtiter plate. A 100 µl of Griess reagent was added to each well, the plate was covered with an aluminium foil and kept at the room temperature for 15 min. Absorbance was measured at 540 nm using a microplate reader (Synergy HTX, multi-mode reader). The levels of nitrite was calculated using the NaNO_2_ standard curve.

Based on the nitrite levels produced by scractched ECs after 12, 24 and 48 h, the 48 h time period was selected for assessing the effect of *M. cordata* extracts on NO production. Cells were treated with 500, 250 and 125 µg/ml of aqueous, acetone and methanolic extract of *M. cordata* at 37 °C 5% CO_2_ incubator and culture supernantants were assessed for nitrite levels using Griess assay.

### Assessment of eNOS and VEGF expression in EA.hy926 cells

Cells were plated in quintuplicates at a concentration of 0.2 × 10^6^ cells/well and was incubated overnight at 37 °C with 5% CO_2_. Scratched cells were treated with 125 µg/ml of methanolic extract of *M. Cordata* and were incubated for 48 h at 37 °C with 5% CO_2_. Cells treated with 1 µg/ml of Allantoin was used as the positive control. Cells were washed and total RNA was extracted using Qiagen RNA extraction kit (RNeasy mini Total RNA) as per the manufacturer’s instructions. cDNA synthesis was done using the GoScript reverse transcription system (Promega, USA) according to the manufacturer’s instructions. The concentration and purity of cDNA were determined using BioSpec Nanospectrophotometer (Shimadzu Corp, Japan) and the A260/A280 ratio of the cDNA sample was 2.0. cDNA (3.7 × 10^6^ ng) was added to a 25 µl reaction mixture, containing dNTPs (10 mM), MgCl_2_ (25 mM), Taq DNA polymerase, and each primer at 10 µM concentration for PCR amplification. Glyceraldehyde 3-phosphate dehydrogenase (GADPH) was used as the control housekeeping gene. Amplification cycles (40 cycles) for eNOS incuded 95 °C for 2 min, 95 °C for 30 sec, 50 °C for 30 sec, 72 °C for 1 min, and 72 °C 5 min. The amplification cycles (40 cycles) for VEGF included 95 °C for 2 min, 95 °C for 30 sec, 57 °C for 30 sec, 72 °C for 1 min, and 72 °C for 5 min. The amplification cycles (35 Cycles) for GADPH included 94 °C for 5 min, 94 °C 30 sec, 56 °C for 30 sec, 72 °C for 40 sec and 72 °C for 5 min. The following sense and anti-sense primers were used respectively; eNOS; 5^’^-ACC CTC ACC GCT ACA ACA T-3^’^ and 5’-GCT CAT TCT CCA GGTGCT TC-3’; VEGF: 5’-ACA CAT TGT TGG AAG AAG CAG CCC-3’ and 5’-AGG AAG GTC AAC CAC TCA CAC ACA-3’; GADPH: 5’−5’-GAA GTT GAA GGT CGG AGT − 3’ and 5’-GAA GAT GGT GAT GGC ATT TC-3’. The PCR products were resolved by electrophoresis on a 2% gel and visualized by Ethidium bromide staining.

### Data analysis

Intensities of PCR bands were quantified using ImageJ software. The expression of eNOS and VEGF gene was normalised to GADPH to calculate the expression of eNOS and VEGF during the process of the closure of the scratch. The results were expressed as the mean ± SEM of at least three independent experiments performed in hexapleticates. Statistical analysis was performed using ONE way ANOVA. Statistical significance was set at *p* value < 0.05.

## Results

### Non-toxic concentrations and effect of Mikania cordata leaf extract on the overall EC function

**Fig. 1:**
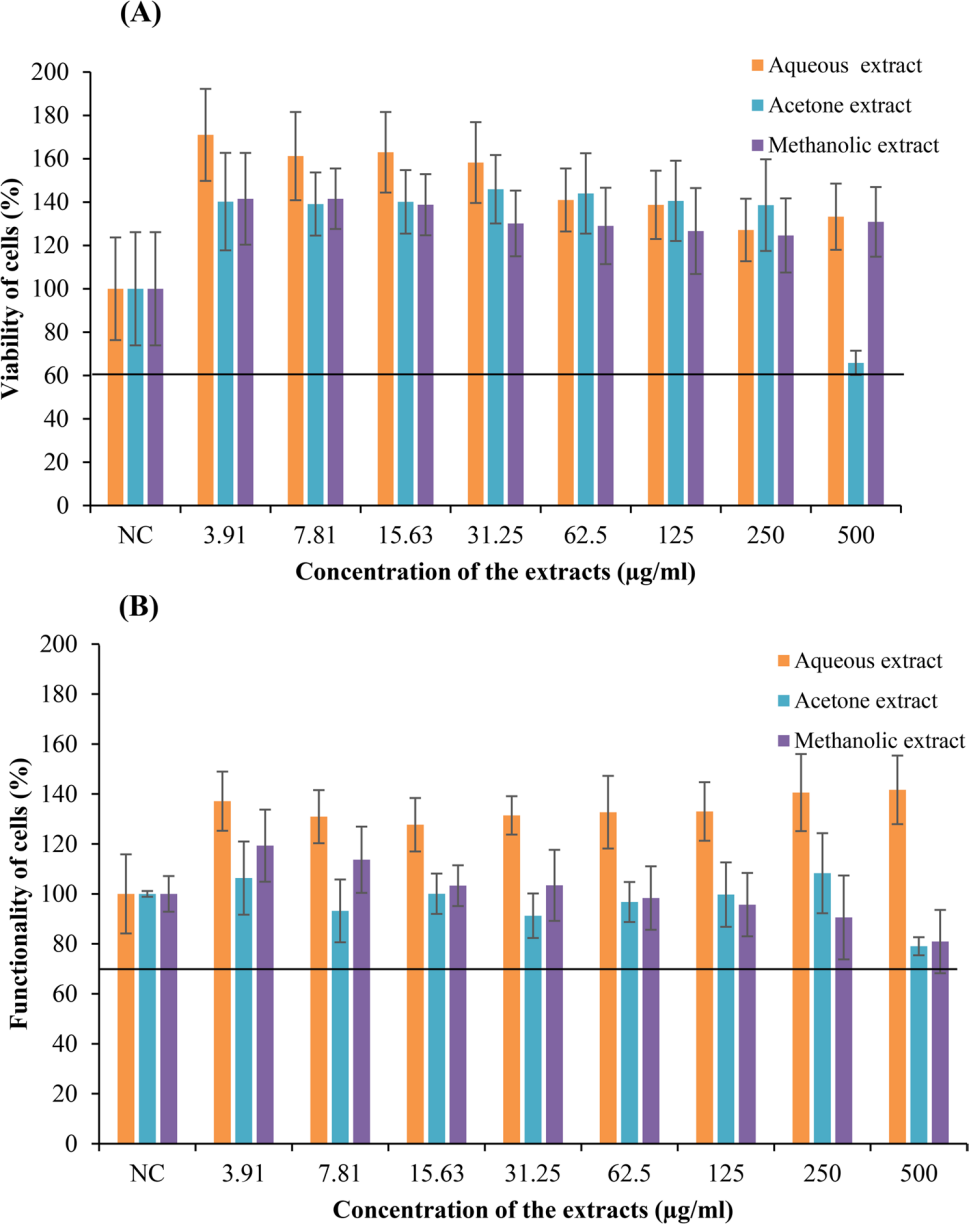
Viability and functionality ECs treated with *M. cordata* leaf extracts for 48 h. **A** Percentage viability of cells determined by SRB assay and (**B**) NADPH oxidase activity of cells determined by MTT assay. Two separate experiments performed in triplicate, and data expressed as mean ± SD (*n*=6). NC – negative control, untreated cells

With decreasing treatment concentrations, an increase in the percentage of viable cells was observed in the cells treated with the leaf extracts of *M. cordata*. The cells treated with concentrations ranging from 3.9 to 500 µg/ml of the aqueous, acetone and methanolic extracts of *M. cordata* demonstrate a steady increase in viability (Fig. 1A). A percentage viability of more than 60% was noticed in cells treated with the aqueous, acetone and methanolic extracts of *M. cordata* at 48 h (*p* < 0.001).

Cells treated with aqueous, acetone and methanolic extracts of *M. cordata* showed an increase in the percentage of NADPH oxidase functioning greater than 70% after 48 h (Fig. 1B). An increase in the percentage activity of NADPH oxidase of cells treated with the methanolic extract of *M. cordata* could be observed with the decreasing concentration of treatment after 48 h. The cells treated with the maximum concentration (500 µg/ml) of the methanolic extract displayed a viability of more than 100% but only 80% functioning. Based on the results of SRB and MTT assays cells treated with 500, 250 and 125 µg/ml of aqueous, acetone and methanolic extracts of *M. cordata* were selected to be used in the scratch assay since a viability and functionality of more than 70% was observed at 48 h in the endothelial cells treated with these particular extracts (*p* < 0.001).

### Effect of M. cordata leaf extracts on EC migration and cell morphology

**Fig. 2:**
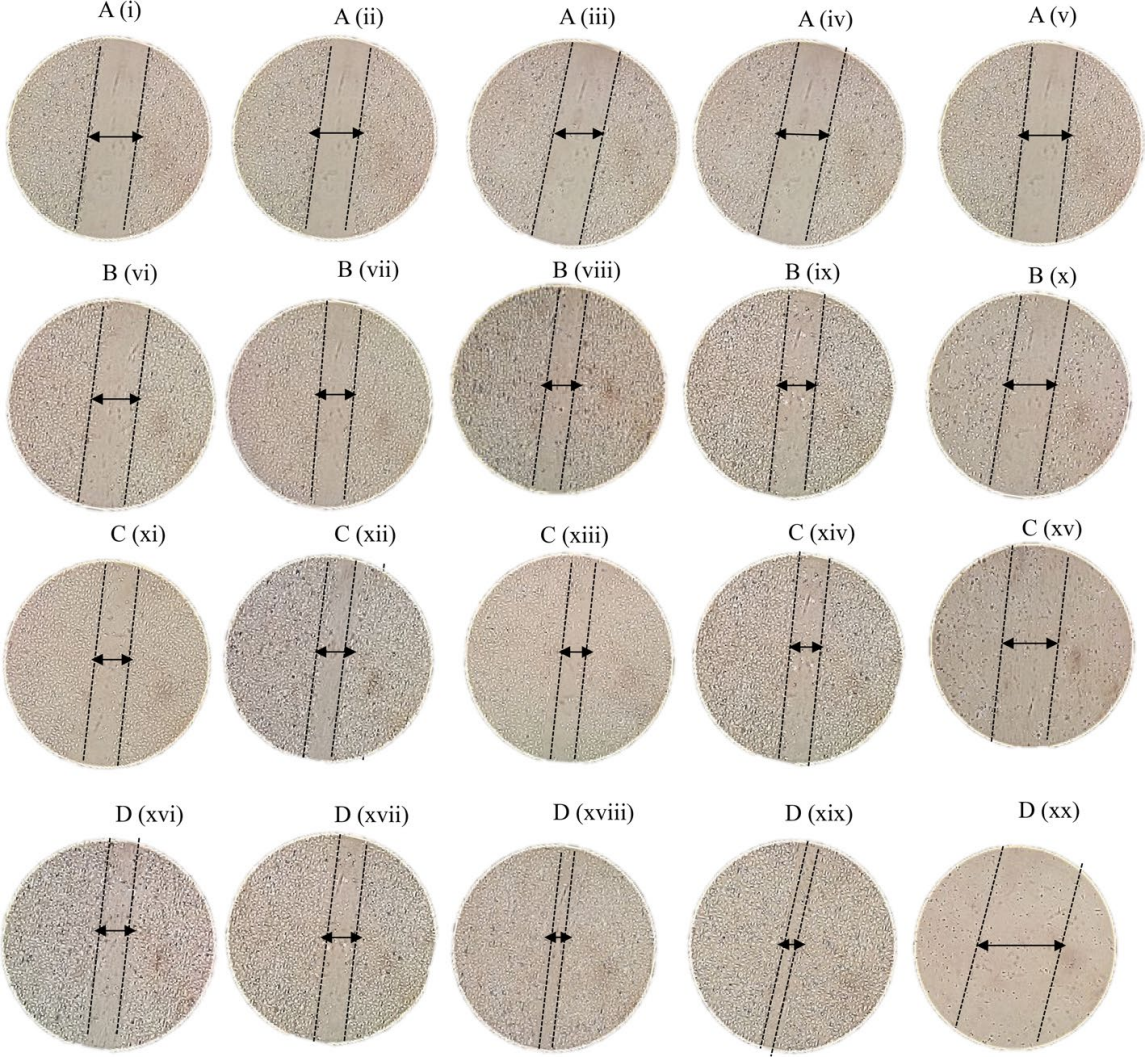
Appearance of ECs treated with the methanolic leaf extract of *M. cordata*. Immediately after treatment (0 h) (**A**), after treatment for 12 h (**B**), 24 h (**C**) and 48 h (**D**) with methanolic extract at 500 µg/ml (i, vi, xi, xvi), 250 µg/ml (ii vii, xii, xvii), 125 µg/ml (iii, viii, xiii, xviii), 1 µg/ml of allantoin (iv, ix, xiv, xix) and scratched cells (v, x, xv, xx). Four separate experiments performed in triplicate, and data expressed as mean ± SD (*n*=6)

**Fig. 3:**
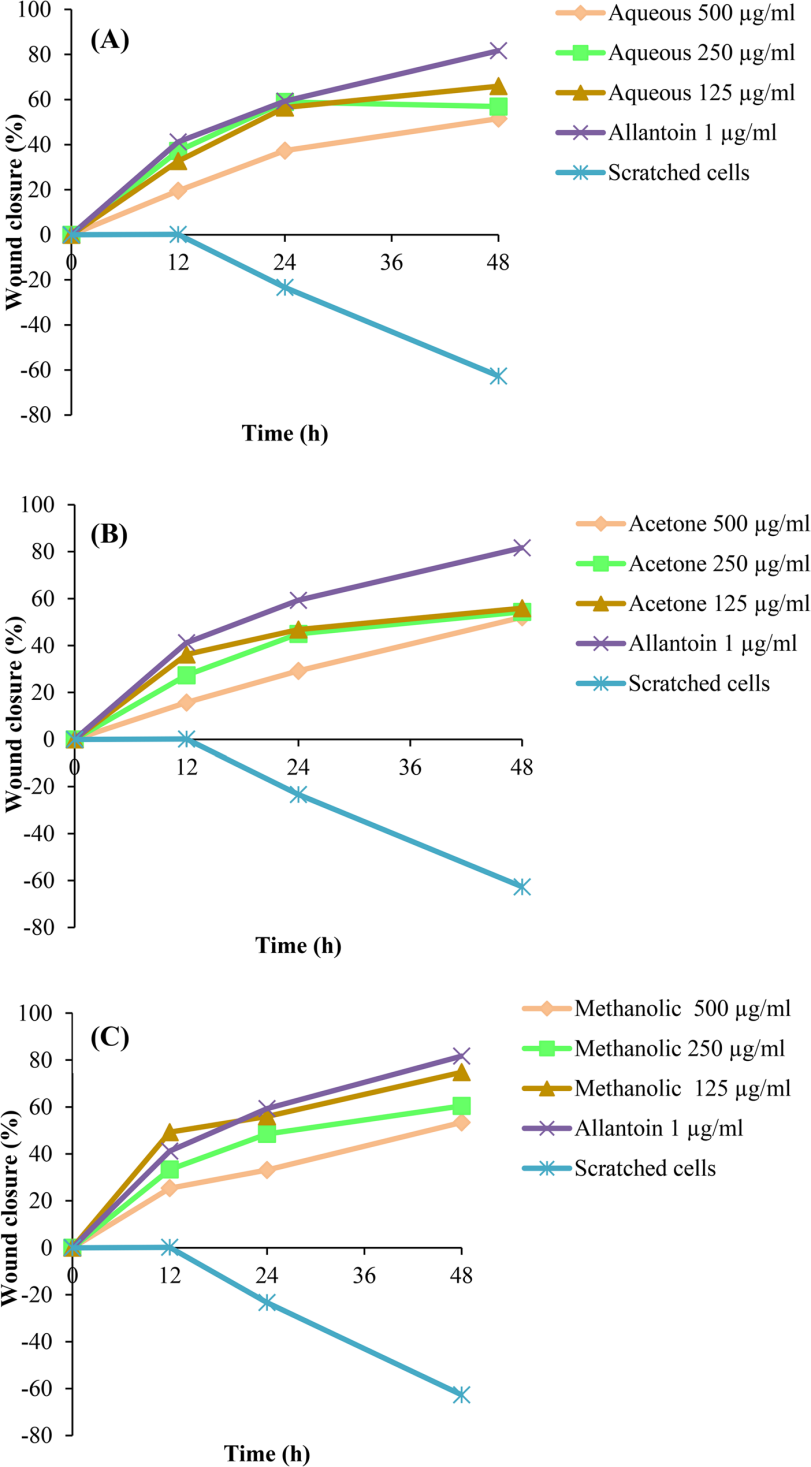
Time dependent ‘gap’ closure of the ECs treated with *M. cordata* extracts, untreated ECs and Allantoin used as the positive control. **A** aqueous extract, (**B**) acetone extract and (**C**) methanolic extract. Four separate experiments performed in triplicate, and data expressed as the mean ± SD (*n*=6)

ECs were treated with 500 µg/ml, 250 µg/ml and 125 µg/ml aqueous extract, acetone (Supplementary material 1 and 2) and methanolic (Fig. 2) extract of *M. cordata* after creating the ‘scratch’/gap and their wound/gap closure was observed at 0, 12, 24 and 48 h. The graphical representation of the ‘gap’ closure of the cells treated with the *M. cordata* leaf extracts and Allantoin is shown in Fig. 3.

At 48 h, the percentage of ‘gap’ closure in cells treated with 125 µg/ml of *M. cordata* methanolic extract is shown in Fig. 2 in comparison to cells treated with 500 µg/ml and 250 µg/ml of *M. cordata* methanolic extract. A ‘gap’ closure percentage of 53% was shown by Ecs treated with 500 µg/ml whereas a gap closure percentage of 60% was shown by cells treated with 250 µg/ml. Cells treated with 125 µg/ml of the methanolic extract *of M. cordata* had a 75% closure rate for ‘gap’. ECs treated with the methanolic extract display a higher percentage of ‘gap’ closure compared to those treated with the aqueous and acetone extracts of *M. cordata* (*p* ≤ 0.001). The percentage of ‘gap’ closure was higher in the cells treated with 125 µg/ml of *M. cordata* methanolic extract, and there was a statistically significant difference between these two values (*p* < 0.001).

The ECs treated with aqueous, acetone (supplementary material 1 and 2), methanolic extracts of *M. cordata* and allantoin at 0, 12, 24 and 48 h (Fig. 2 Ai-iv, Bvi-ix, Cxi-xiv, and Dxvi-xix) remained polygonal, elongated and spindle shaped refelecting their endothelial nature. However, there were changes observed in cell morphology of ECs 12 h after scratching (Bx). At 24 (Cxv) and 48 h (Dxx), the ECs appeared to have fragmented cell membranes indicating loss of membrane reflecting characteristics associated with stress response.

### Effect of M. cordata leaf extracts on production of nitric oxide

**Fig. 4:**
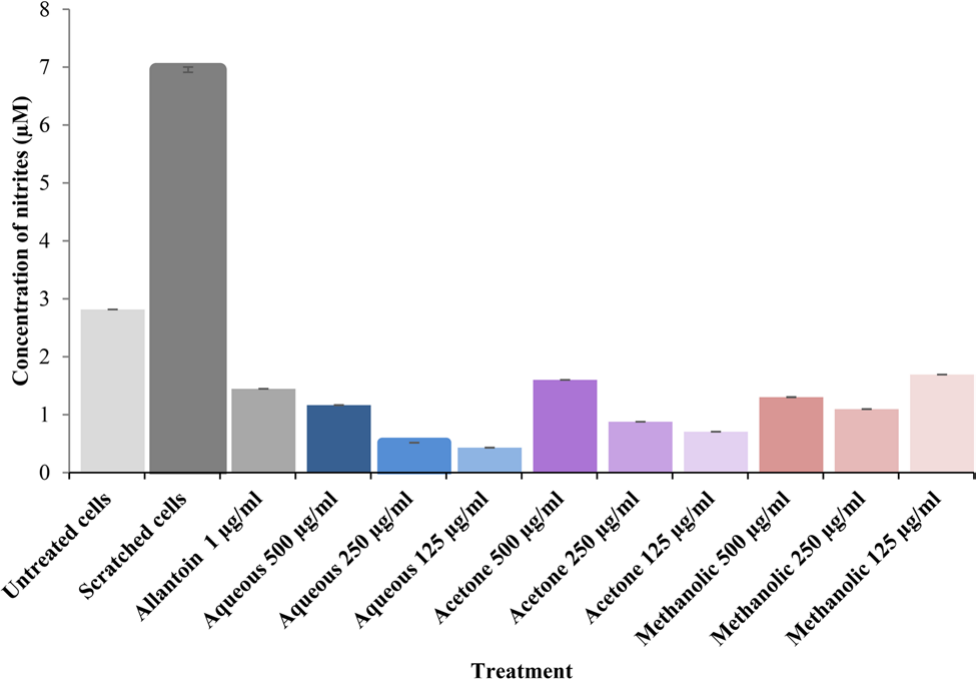
Effect of *Mikania cordata* leaf extracts on production of nitric oxide by untreated cells, scratched cells, treated with 1 µg/ml of allantoin, 500, 250 and 125 µg/ml of aqueous, acetone and methanolic extracts of *M. cordata*. On four separate studies, each assay was carried out in triplicate, and measurement data were expressed as the mean ± SD (*n*=6)

After 48 h incubation, the scratched cells produced a significantly high level of nitric oxide as measured by the nitrite level in culture supernant (7 ± 0.045 µM) compared to unscratched cells (2.8 ± 0.001 µM, *p* < 0.001) (Fig. 4). The nitrite levels measured in culture supernatants of the scratched cells after 12 and 24 h were less than 1 µM (data not shown). Therefore, the 48 h incubation was selected for the treatment with *M. cordata* leaf extracts. A significant decrease in the levels of nitrites was observed in the scratched, cells treated with aqueous, acetone and methanolic leaf extracts of *M. cordata* compared to that of the scratched, untreated cells (*p* < 0.001). Allantoin treatment (1 µg/ml) had significantly decreased production of nitric oxide (1.4 ± 0.001 µM, *p* < 0.001) compared to that of the scratched cells and methanolic extract (125 µg/ml) also showed a similar effect having 1.7 ± 0.001 µM nitrites. However, the nitrite levels observed after 48 h in culture supernantants in the scractched, cells treated with the extracts were lower compared to that in unscratched, untreated cells.

### Expression of eNOS and vascular endothelial growth factor in wound healing

**Fig. 5:**
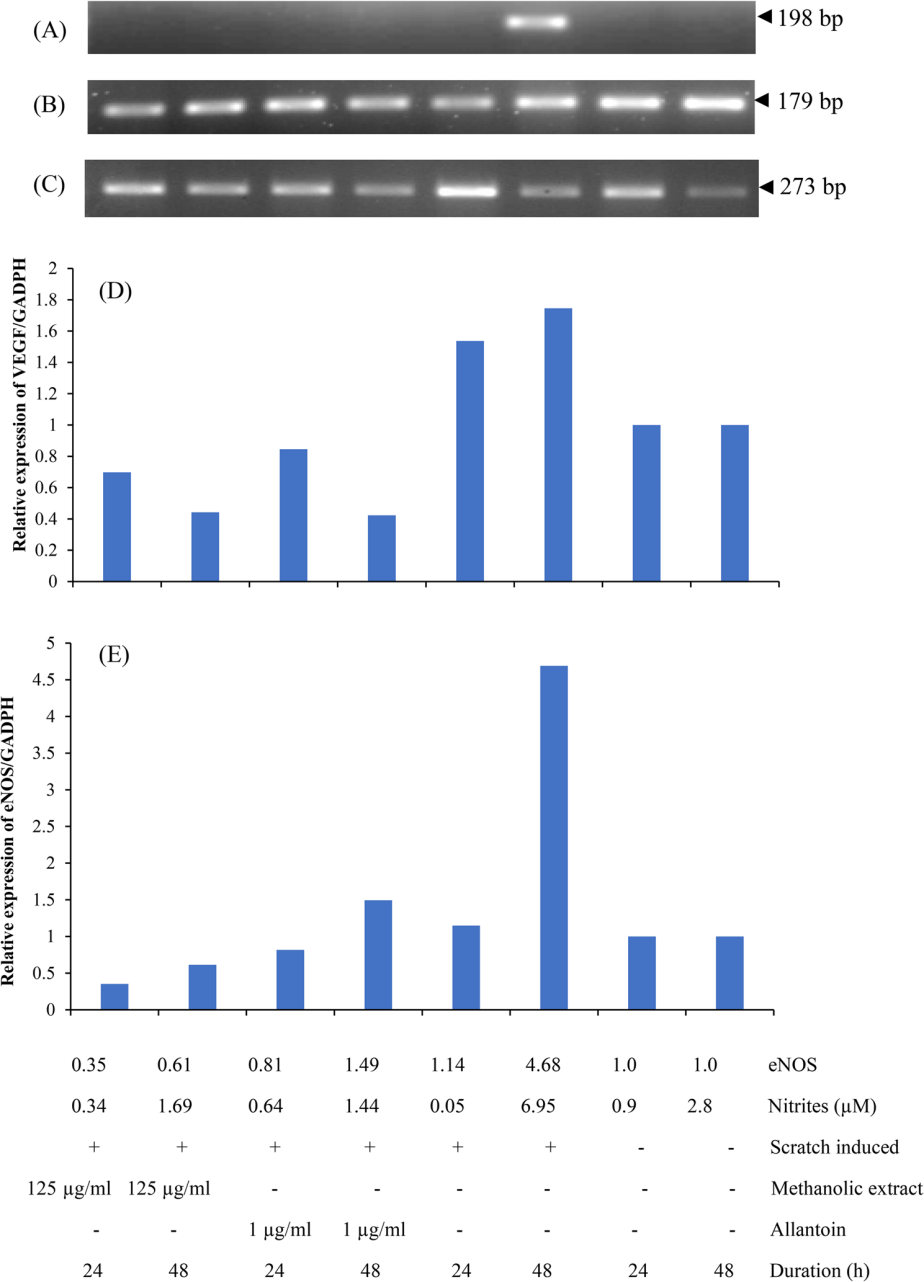
Expression of eNOS and VEGF in the scratch assay using EA.hy926 cells. RT-PCR analysis for the mRNA expression of eNOS gene (**A**), VEGF gene (**B**) and GAPDH gene (**C**) used as a control. Relative expression VEGF/GAPDH (**D**) and eNOS/GAPDH (**E**). Total RNA was extracted from treated cells in pentaplicates and the eNOS, VEGF and GAPDH mRNA expression were tested by RT-PCR in two repeat experiments (*n* = 6)

Conventional PCR was carried out to investigate the expression of eNOS, VEGF and GADPH in the endothelial cells during an inflammation. The agarose gel electrophoresis image of the expression of these genes are shown in Fig. 5.

The amount of VEGF increases during the formation of wound and comes to undetectable levels once the would is healed completely. The highest expression of VEGF is observed after 48 h of the scratched ECs (1.74) whereas these VEGF levels decreases to 0.44 and 0.42 in the scratched ECs treated with 125 µg/ml of methanolic extract of *M. cordata* and 1 µg/ml of allantoin (Fig. 5D). A correlation between the levels of nitrites and expression of eNOS can be observed (Fig. 5E). The highest level of nitrites (6.95 µM) can be observed in the scratched, untreated cells at 48 h whereas the highest expression of eNOS (4.68) is also observed in the same.

## Discussion

Traditional medicinal practices and substances have been used because of their therapeutic benefits, accessibility, affordability, and relatively cheap cost in order to promote the ideal conditions for skin regeneration and prevent the failure of the healing process. The terms “alternative” and “complementary” medicine are also used to describe traditional medicine. These treatments incorporate techniques, materials, and knowledge from different countries and utilize plant substances that can be found in a variety of situations [24].

The plant *M. cordata* has been used for its antibacterial, anti-inflammatory, and wound-healing properties. *M. cordata*, also known as “wathu paalu,” has been used widely in traditional medicine in Sri Lanka for a very long time for its beneficial effect on the healing of wounds. *M. cordata* is thought to be a source of bioactive compounds that should be further researched [18].

For the purpose of producing high-quality study results, it is essential to first prepare medicinal plants for trial. Before starting the required biological testing, the bioactive components must first be extracted, and their quantity and quality should be evaluated. It is feasible to prepare medicinal plants for research or to extract and process them for use directly as herbal or conventional medicine [25]. The concept of preparing a medicinal plant for experimental purposes comprises the timely and proper collection of the plant, professional authentication, optimal drying, and grinding.

The current study is an in vitro investigation into the effects of *M. cordata* leaf extracts from three different solvents with various polarities on the capacity to heal wounds. EA.hy926 is a type of adherent macrovascular endothelial cell line. The effect of the *M. cordata* leaf extracts on wound healing capacity was investigated. This EA.hy926 cell line was chosen primarily due to its ability to participate in the fundamental stages of wound healing, including angiogenesis, haemostasis/thrombosis, and inflammation [26].

With over 430 species, mostly found in tropical areas, the genus *Mikania* is the largest of its kind in the *Asteraceae* tribe. The chemical constituents of about 12% of *Mikania* species have been investigated. Various classes of chemicals which have been linked to the pharmacological actions of this plant have been previously identified from different parts of *Mikania.* Coumarins and their derivatives, sesquiterpenes, sesquiterpene lactones, diterpenes, terpenoids, and flavonoids are the principal categories found [27]. The pharmacological activity of diterpenes such as benzzoylgrandifloric acid and kaurenoic acid has also drawn interest. The genus *Mikania* is home to a large number of sesquiterpene compounds, including gemacrene D, isocomene, and γ-humulene which have been found in about 15% of *Mikania* species. The *Mikania* genus contains the sesquiterpenes germacrene D, isocomene, and γ-humulene, which have a variety of pharmacological actions such as antioxidant, antibacterial, anti-inflammatory, and anticancer properties [28].

During sequential extraction, the chosen solvent is added in ascending polarity order, beginning with the least polar, and ending with the most polar solvent. The main purpose of carrying out a sequential extraction is that different compounds in the leaf extract can be extracted when using solvents with different polarities. Compared to acetone, methanol has a higher polarity. Acetone has a polarity rating of 0.355Å, making it a less polar solvent. Methanol has a polarity of 0.762Å [24]. By employing solvents with various degrees of polarity, several chemicals that are present in the leaves extracts of *M. cordata* can be extracted.

Before investing in clinical research, preclinical testing is essential for identifying suitable candidates. The use of grown cells for the initial preclinical screening of potential therapeutic medications has become commonplace because they can be selected to reproduce the disease of interest or its accompanying metabolic abnormalities. The accuracy and dependability of the outcomes from the in vitro cytotoxicity tests employed in the initial stages of preclinical research may determine whether a medication candidate is successful in moving further into the development process [25].

Many of the phytochemicals in *M. cordata* leaf extract that are found in acetone may not be present in the methanolic or aqueous extracts and vice versa. When cells are exposed to these extracts, some of the chemicals included therein may be hazardous as well as non-toxic. As a result, the 3-(4,5-dimethylthiazol-2-yl)−2,5-diphenyltetrazolium bromide) tertazolium reduction assay (MTT) and sulforhodamine B assay (SRB) were conducted.

SRB assay and MTT assay were carried out to study the viability and functionality of cells respectively. EA.hy926 cells were treated with different concentrations of aqueous, acetone, and methanolic extracts of *M. cordata* ranging from the concentrations of 3.905, 7.81, 15.63, 31.25, 62.5, 125, 250, and 500 µg/ml. The cells treated with the 500 µg/ml of aqueous extract *M. cordata* for 48 h showed a percentage viability of 133% and a functionality of 141%, respectively. This depicts the fact that aqueous extracts are not toxic to cells even at their highest concentration and that they may have an impact on cell viability and functionality. Cells treated with 500 µg/ml acetone and methanolic extracts of *M. cordata* at 48 h showed a percentage viability of 65% and 130% and a functionality of 79% and 81%. This could be because although cells that are still alive are considered viable, this does not always mean that they are operating correctly. Even though they are viable with intact membranes, some cells may not be able to perform their regular functions such as metabolism, signaling, or stimulus response, with the addition of an extract. Due to this reason, a decrease in the functionality of the ECs treated with methanolic extract could be observed with the increase of percentage viability. Also, with the decreasing concentrations of the treatment, an increase in the percentage viability and functionality of the cells was observed. In a study that was carried out using methanolic extract of a different plant extract, similar results have been observed where the methanol extract depicted cytotoxic activity in a dose-dependent manner [29].

The scratch assay is the favoured method for studying cell migration due to its low cost and simple experimental design. The scratch assay is an in vitro method for evaluating how cellular and molecular processes impact cell migration. Before being employed in clinical trials, pharmaceutical substances can also be evaluated using the test. Following skin injury, cell migration and proliferation are crucial for effective wound healing. Recent studies have demonstrated that plant-derived chemicals encourage cell migration and proliferation.

A skin-active ingredient called allantoin possesses keratolytic, moisturizing, soothing, and anti-irritant properties. Additionally, it speeds up the renewal of epidermal cells and promotes faster wound healing. By encouraging the regeneration of injured epithelium and boosting epidermal cell proliferation, allantoin improves wound healing. Allantoin is utilized as a positive control in most studies to evaluate in vitro wound healing since it is known to stimulate wound healing [30].

In the current study, the scratch assay was carried out in order to study the effect of *M. cordata* leaf extracts on wound healing. The cells were treated with 500, 250, and 125 µg/ml aqueous, acetone, and methanolic extracts of *M. cordata*. The positive control used was allantoin. The concentrations of allantoin used were 50, 10, 5, and 1 µg/ml. According to published accounts, the aforementioned concentrations of allantoin allowed for the complete closure of the lesion [30]. Therefore, the above concentrations of allantoin were employed in this study to determine at which concentration the wound closure would be at its highest. The cells treated with 1 µg/ml of allantoin showed the highest percentage of wound closure. Therefore, the cells were treated with 1 µg/ml of allantoin throughout the experiments.

Using ImageJ software, the distance of wound closure was calculated based on the migration of cells at 0, 12, 24, and 48 h. All three extracts of *M. cordata* were tested on the scratched ECs at 500, 250, and 125 µg/ml concentrations, and the best closure was noted in the scratched Ecs treated with 125 µg/ml by 48 h. Ecs treated with 125 µg/ml of aqueous extract of *M. cordata* depict gap closure of 66% at 48 h when compared to that of 500 µg/ml and 250 µg/ml (52% and 57%) of aqueous extract of *M. cordata* as shown in Fig. 3A. A gap closure of 56% was observed in Ecs treated with 125 µg/ml acetone extract of *M. cordata*. The ECs treated with 500 µg/ml and 250 µg/ml of acetone extract of *M. cordata* showed a gap closure of 52% and 54% as shown in Fig. 3B. Methanolic extract treated with 125 µg/ml resulted in the highest percentage of closure (75%) among the three extracts, and the cells treated with 1 µg/ml of allantoin (positive control) closed the gap by 82% at 48 h. A gap closure was not observed in the scratched untreated ECs. A higher gap closer was observed in the aqueous and methanolic extracts of *M. cordata* when compared to that of the acetone extact. This can be because, the therapeutic activities of *M. cordata* may be attributed to a variety of water-soluble phytochemicals found in its aqueous extracts. Since water is a polar solvent, it would mostly draw out polar and water-soluble substances from the plant [31]. These substances would have antibacterial, anti-inflammatory, and antioxidant qualities. In a study utilizing a different plant extract, it was shown that the methanolic extract encouraged keratinocyte and fibroblast migration and increased the expression of genes involved in wound healing [32]. Further in a study conducted in 2019 by Truong and his colleagues, methanol was found to be the most efficient solvent for the extraction, producing the highest yield and the highest content of phenolic compounds, flavonoids, and terpenoids. Additionally, it is known to have strong antioxidant and in vitro anti-inflammatory action, as well as membrane stability [33]. However, as there is limited data on the phytochemical compounds present in these extracts of *M. cordata*, additional chemical analysis employing chromatography or spectrometry is required to identify the entire range of phytochemicals found in methanolic, acetone, and aqueous extracts of *M. cordata* which contributes to the process of effective wound healing. These findings may demonstrate that methanolic extract of *M. cordata* is more preferable to its other two extracts in terms of its ability to speed up the healing of wounds.

Nitric oxide (NO), a low molecular weight and highly diffusible gaseous molecule that regulates numerous physiological and pathological processes and promotes endothelial cell proliferation, migration, and vasodilation. The Griess test is the method for NO quantification that has been published the most frequently. Nitric oxide functions as an inflammatory regulator and endogenous antibacterial agent which drives its use to promote wound healing [34]. It uses spectrophotometry to quantify nitrite (NO_2_), a stable consequence of NO autoxidation. This method is simple, reasonably priced, and widely available in the market [35].

An essential endogenous gasotransmitter in wound healing is nitric oxide. Nitric oxide functions as an inflammatory regulator and endogenous antibacterial agent, which drives its use to promote wound healing [35]. The EC treated with the three *M. cordata* extracts aqueous, acetone, and methanol showed a continuous increase in the production of nitrite levels up to 48 h. As noted in the literature, an increase in nitrite levels can be seen during an inflammation, and with the healing of a wound, the nitrite level stabilizes over time. However, in this study the nitrite levels were significantly lower in the treated Ecs than the scratched cells (*p* < 0.001). A significant decrease in the levels of nitrites in Ecs treated with methanolic extract was observed when compared to that of the scratched untreated cells (*p* < 0.001). These results may suggest that Ecs treated at a concentration of 125 µg/ml of all the three extracts of *M. cordata* had induced the production of NO, which potentially would have contributed to wound healing observed. A similar trend in the levels of nitrite can be observed in the cells treated with 1 µg/ml of allantoin to that of the cells treated with 125 µg/ml of methanolic extract of *M. cordata*. This may provide evidence that the methanolic extract of *M. cordata* is more potent in healing when compared to the activity of aqueous and acetone extracts.

The expression of nitrites on the healing of the wound was investigated by conventional PCR. The gene expression for eNOS was studied. The expression of eNOS was analysed using the ImageJ software. Tiny peaks were found when the agarose gel electrophoresis of eNOS expression was examined using the ImageJ software. This shows that eNOS was present in the sample, albeit in very small amounts, and these bands would have observed with a higher template DNA concentration. The specific band for eNOS was observed only in the scratched untreated cells. The scratched untreated cells exhibit a very significant increase in the expression of eNOS (4.6), and the band may have only been visible in the scratched untreated cells and not in the other samples since, according to the Griess test results, the highest amount of nitrite released was observed in the scratched untreated cells. No bands were found in any of the other samples, which may be explained by the extremely low expression of nitrites in any of them, according to the analysis of the Griess assay results. After 24 and 48 h of treatment with the methanolic extract of *M. cordata*, eNOS expression of 0.3 and 0.6 was observed in this study. Cells treated with allantoin also show similar eNOS expression at 24 and 48 h (0.8 and 1.4), which is similar to the expression of ECs at its normal physiological state, proving the fact that the expression of eNOS is important for the process of wound healing. This may demonstrate the potency of methanolic leaf extract of *M. cordata* for wound healing.

The expression of VEGF on the healing of wounds was investigated by conventional PCR. The expression levels of VEGF were examined in cells treated for 24 and 48 h with 125 µg/ml methanolic extract of *M. cordata*, Allantoin, scratched cells, and in untreated cells. Proangiogenic factors are molecules that encourage angiogenesis, or the growth of new blood vessels. Fibroblast growth factor (FGF), vascular endothelial growth factor (VEGF), and other signalling molecules are a few examples [36]. These factors are important in enhancing blood flow to the injured area, which brings in oxygen and nutrients needed for healing where these factors are essential for tissue repair following an injury. Proangiogenic factors are released by the body to promote the formation of new blood vessels following an injury. The concentration of these proangiogenic substances rises and peaks at a specific stage of the healing process. Proangiogenic factors encourage the development of new capillaries in the damaged tissue. “Maximum capillary content” would be reached when the wounded area’s capillary network has fully developed and the highest volume of blood is delivered to the tissue. Proangiogenic factor levels begin to decline following the peak of angiogenesis, or the formation of new blood vessels. The body no longer needs to maintain high levels of these factors once sufficient blood vessels are formed. Their concentrations fall to very low or nearly undetectable levels. This shows that there is less need for more angiogenesis because the tissue has already been repaired [36].

The band’s intensities are inversely correlated with the proteins produced by the genes expressed in the sample. Lesser band intensities result in lower amounts of VEGF expression, while higher band intensities result in more gene expression. In comparison to the bands that manifested in the cells treated with 125 µg/ml of methanolic extract and 1 µg/ml of allantoin, the intensity of the band in the scratched cell was higher.

After 48 h, the cells exposed to 125 µg/ml methanolic leaf extract of *M. cordata* displayed a decrease in VEGF expression when compared to that at 24 h. The VEGF expression was down-regulated when compared to that of scratched cells. Additionally, VEGF was down-regulated in the Allantoin treated cells (positive control). At 48 h, the scratched cells showed a greater expression of VEGF (1.74). This shows that there is no wound healing taking place and that the inflammation is growing over time in scratched cells. Additionally, the cells treated with the methanolic extract of *M. cordata* and allantoin displayed decreased VEGF expression, indicating a higher percentage of wound closure. GADPH served as the housekeeping gene in our investigation [37]. GADPH was expressed in all of the samples, demonstrating that even with the addition of treatment, the normal physiological function is still being carried out. However, after 48 h of treatment, an increase in the expression of GADPH was seen in the cells treated with a methanolic extract of *M. cordata* and allantoin, suggesting that the physiological activity of the cells is increased. The methanolic extract of *M. cordata* performs similarly to that of the positive control, as evidenced by the expression of the same levels of GADPH as the positive control.

However, further studies are needed to be carried out to find the active compounds present in these extracts which aids in the process of wound healing. Other investigations of a similar nature can be conducted *in vivo*, and this particular *M. cordata* extract may then be employed in clinical trials. Studies based on HPLC can also be carried out in order to pinpoint the precise chemical present in the methanolic extract *of M. cordata*. The development of pharmacogenetic drugs for the treatment of chronic wounds can be facilitated by identifying the specific compound contained in the *M. cordata* leaf extract. Also, evaluation of the dermal and oral toxicity of these phytoproducts and *in vivo* studies to evaluate the efficacies of these plant extracts should also be carried out in the future. This study is a pioneering effort that will open the door to the identification of numerous additional *M. cordata* compounds and properties that will be crucial in the creation of pharmacological treatments for wound healing.

## Conclusion

In conclusion, it can be claimed that the methanolic leaf extract of *M. cordata* may contribute to promote wound angiogenesis.

These findings support the notion that *M. cordata* may have therapeutic benefits for the treatment of wounds and may represent a viable source for the extraction of natural chemicals that promote wound healing. The use of *M. cordata* in wound healing may be ethnopharmacologically validated by the present study.

## Supplementary Information

Supplementary Material 1.

Supplementary Material 2.

Supplementary Material 3.

Supplementary Material 4.

## Data Availability

This published article [together with its supplemental information files] contains all of the data created or analyzed during this investigation.

## Abbreviations

Aq: Aqueous
Met: Methanolic
Ace: Acetone
M.: *Mikania*
All: Allantoin
VEGF: Vascular Endothelial Growth Factor
EC: Endothelial cells
