# Supplementary material for: In vitro effect of Mikania cordata leaf extracts on wound healing
Source: BMC Complement Med Ther. 2025 Oct 9;25:366. doi: 10.1186/s12906-025-05110-7 (PMC12512523; doi:10.1186/s12906-025-05110-7)
Supplement: Supplementary file 1 — Supplementary Material 1. [file 12906_2025_5110_MOESM1_ESM.pdf]

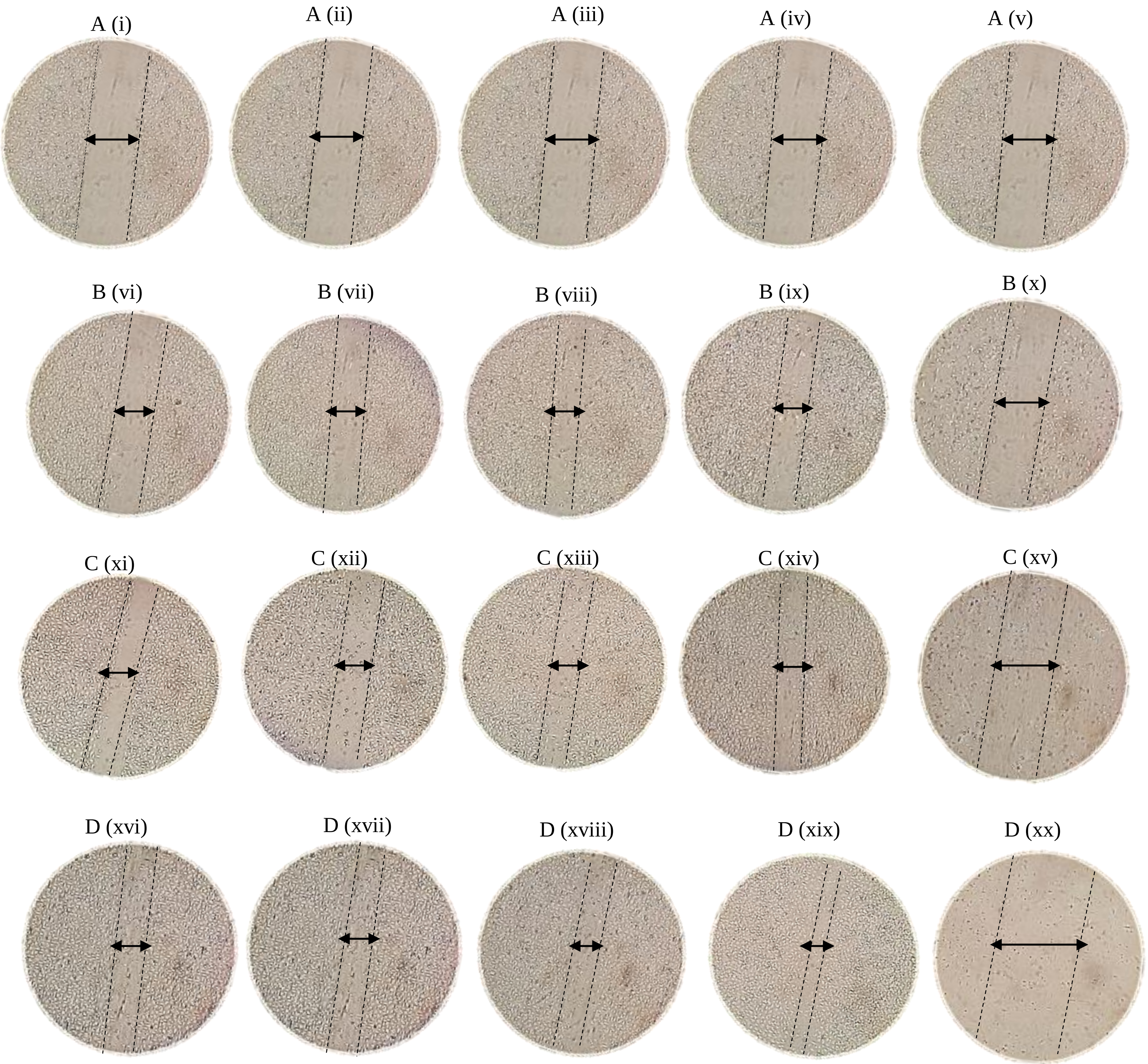

Fig. 6 Appearance of ECs treated with the aqueous leaf extract of *M. cordata*. Immediately after treatment (0 h) (A), after treatment for 12 h (B), 24 h (C) and 48 h (D) with aqueous extract at 500 µg/ml (i, vi, xi, xvi), 250 µg/ml (ii vii, xii, xvii), 125 µg/ml (iii, viii, xiii, xviii), 1 µg/ml of

allantoin (iv, ix, xiv, xix) and scratched cells (v, x, xv, xx). Four separate experiments performed in triplicate, and data expressed as mean ± SD (n=6).
