# Supplementary material for: In vitro effect of Mikania cordata leaf extracts on wound healing
Source: BMC Complement Med Ther. 2025 Oct 9;25:366. doi: 10.1186/s12906-025-05110-7 (PMC12512523; doi:10.1186/s12906-025-05110-7)
Supplement: Supplementary file 2 — Supplementary Material 2. [file 12906_2025_5110_MOESM2_ESM.pdf]

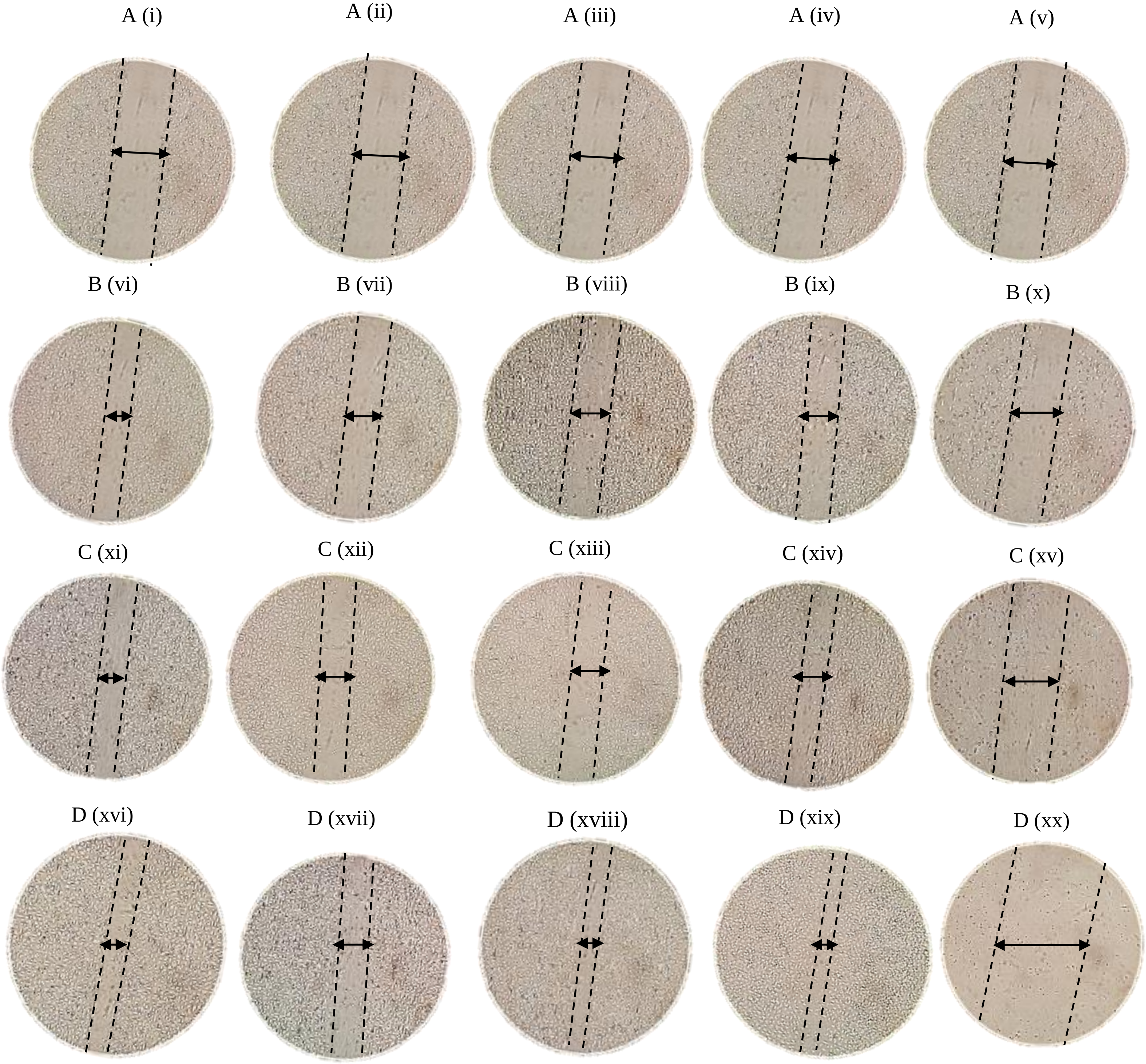
A (i)
A (ii)
A (iii)
A (iv)
A (v)
B (vi)
B (vii)
B (viii)
B (ix)
B (x)
C (xi)
C (xii)
C (xiii)
C (xiv)
C (xv)
D (xvi)
D (xvii)
D (xviii)
D (xix)
D (xx)

Fig. 7 Appearance of ECs treated with the acetone leaf extract of *M. cordata*. Immediately after treatment (0 h) (A), after treatment for 12 h (B), 24 h (C) and 48 h (D) with acetone extract at 500 µg/ml (i, vi, xi, xvi), 250 µg/ml (ii vii, xii, xvii), 125 µg/ml (iii, viii, xiii, xviii), 1 µg/ml of allantoin (iv, ix, xiv, xix) and scratched cells (v, x, xv, xx). Four separate experiments performed in triplicate, and data expressed as mean ± SD (n=6).
