# Supplementary material for: In vitro effect of Mikania cordata leaf extracts on wound healing
Source: BMC Complement Med Ther. 2025 Oct 9;25:366. doi: 10.1186/s12906-025-05110-7 (PMC12512523; doi:10.1186/s12906-025-05110-7)
Supplement: Supplementary file 3 — Supplementary Material 3. [file 12906_2025_5110_MOESM3_ESM.pdf]

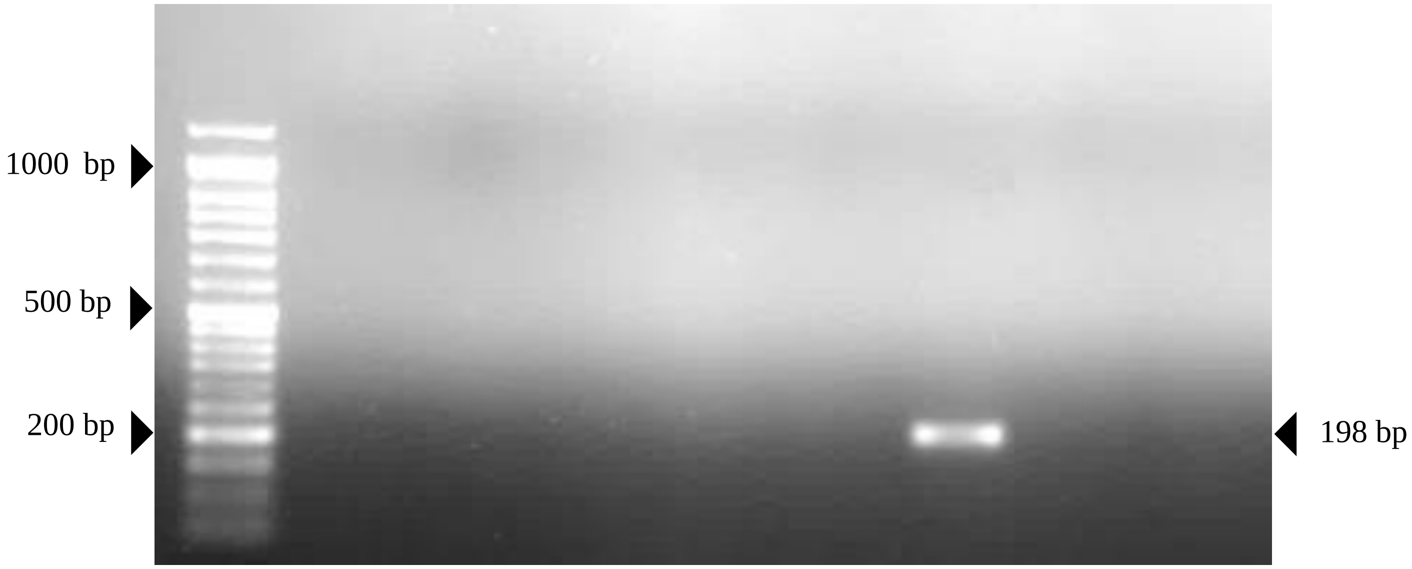

Fig. 8 Expression of eNOS in the scratch assay using EA.hy926 cells.

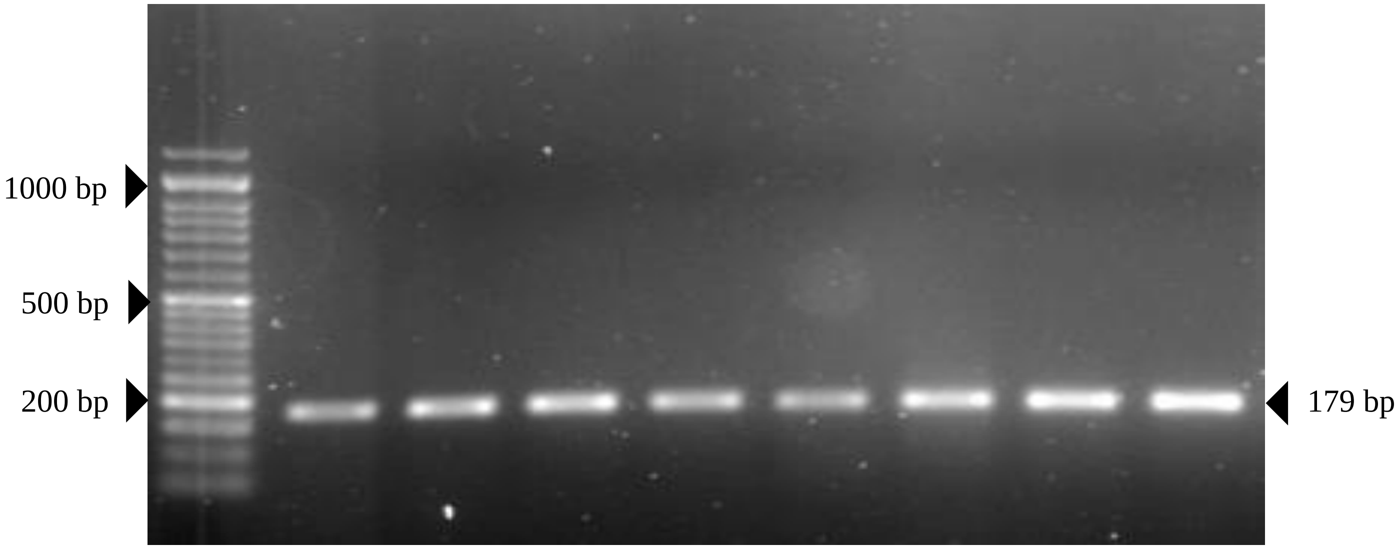

Fig. 9 Expression of VEGF in the scratch assay using EA.hy926 cells.

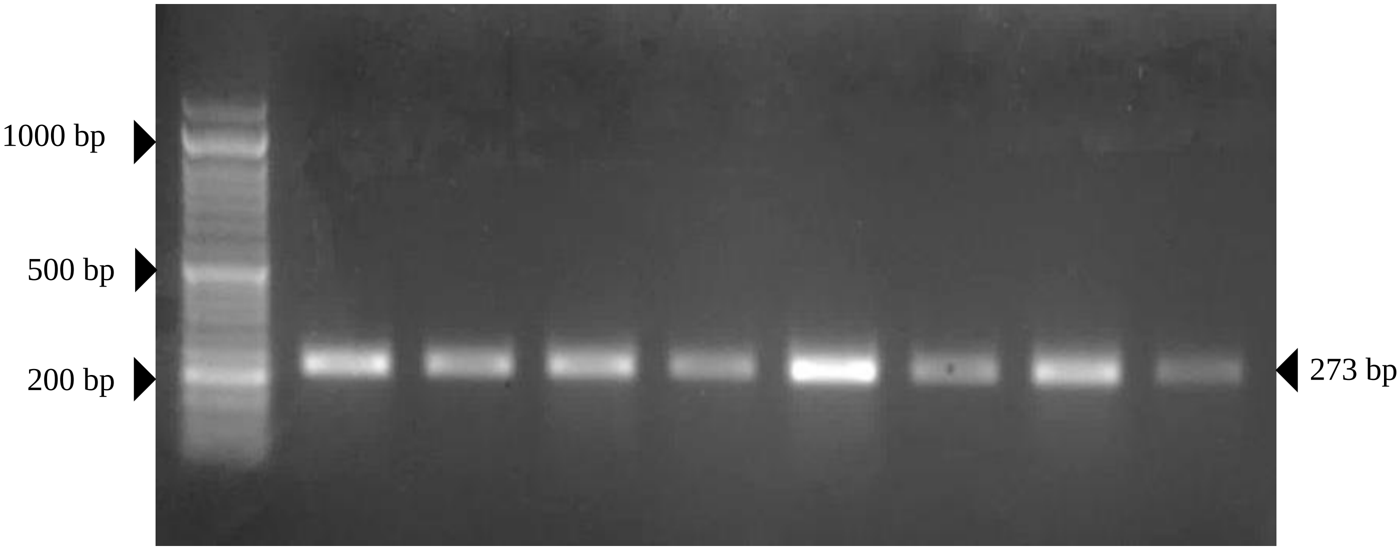

Fig. 10 Expression of GADPH in the scratch assay using EA.hy926 cells.
